# Environmental considerations and bioremediation applications of the metal-, nitrate-, and organohalide-respiring bacterium *Trichlorobacter lovleyi* (formerly *Geobacter lovleyi*)

**DOI:** 10.1128/aem.00563-26

**Published:** 2026-06-03

**Authors:** Kelsie R. Herzer, César I. Torres, Rosa Krajmalnik-Brown, Anca G. Delgado

**Affiliations:** 1 Biodesign Swette Center for Environmental Biotechnology, Arizona State University 7864, Tempe, Arizona, USA; 2 School of Sustainable Engineering and the Built Environment, Arizona State University 310018, Tempe, Arizona, USA; 3 School for Engineering of Matter, Transport and Energy, Arizona State University, Tempe, Arizona, USA; 4 Biodesign Center for Health Through Microbiomes, Arizona State University 7864, Tempe, Arizona, USA; The Pennsylvania State University, University Park, Pennsylvania, USA

**Keywords:** dissimilatory nitrate reduction of ammonium, extracellular electron transfer, organohalide respiration, *Trichlorobacter lovleyi*, *Geobacter lovleyi*, bioremediation, *Dehalococcoides*

## Abstract

*Trichlorobacter lovleyi* (formerly *Geobacter lovleyi*) is a gram-negative bacterium that can couple the oxidation of acetate or other small organic acids with the reduction of soluble and insoluble electron acceptors, such as chlorinated solvents, heavy metals, and nitrate. Evidence has shown that *T. lovleyi* plays a direct role in nitrogen, iron, and carbon cycling, and its versatile metabolism can be leveraged for environmental biotechnology applications. However, the contributions of *T. lovleyi* to treatment of contaminants at groundwater sites or electron transfer in bioelectrochemical systems have been largely overlooked. This minireview examines the genetic and metabolic features of *T. lovleyi* for dissimilatory reduction of nitrate to ammonium, extracellular electron, and organohalide-respiration, highlighting unique and conserved features relative to other genera in *Geobacterales.* We highlight applications of *T. lovleyi* in bioremediation of contaminated environments and identify knowledge gaps to fully leverage the metabolic potential of this bacterium. We feature *T. lovleyi*’s ability to produce the important vitamin B_12_ cofactor and the syntrophic partnership it establishes with cobalamin-scavenging, obligate organohalide-respiring bacteria, particularly in environments where the concentration of chlorinated solvents poses toxicity challenges to the groundwater microbiome. We conclude that *T. lovleyi* has a meaningful, multi-faceted, but often neglected, contribution to groundwater and soil bioremediation. We hope this minireview will prompt researchers and bioremediation practitioners to more closely monitor *T. lovleyi* during reductive dechlorination enrichment efforts and bioremediation applications at sites contaminated with organohalogens, uranium, or nitrate.

## INTRODUCTION

*Trichlorobacter lovleyi* (*T. lovleyi*) is a gram-negative bacterium in the *Thermodesulfobacteriota* phylum that can couple the oxidation of acetate or other small organic acids with the reduction of soluble and insoluble electron acceptors, such as chlorinated solvents and heavy metals. *T. lovleyi*, originally described as *Geobacter lovleyi* (*G. lovleyi*), was named in honor of Derek R. Lovley, microbiologist and professor at the University of Massachusetts Amherst. *T. lovleyi* was isolated from a noncontaminated aquatic sediment (report published in 2006) (1) but has since been detected as an active microbial species in ferric iron-reducing and nitrate-reducing environments (2–7). *T. lovleyi* is also part of microbial communities of subsurface contaminated with chlorinated organic solvents, petroleum hydrocarbons, arsenic, or heavy or radioactive metals, anaerobic digesters in wastewater treatment plants, and constructed wetlands (4, 8–18). *T. lovleyi* is an organohalide-respiring bacterium and a prominent member in enrichment cultures used for bioaugmentation applications at groundwater sites contaminated with chlorinated ethenes (19–21). To date, *T. lovleyi* is the only bacterium reported to reduce the chlorinated solvent, tetrachloroethene (PCE), and the radioactive metal, hexavalent uranium (U(VI)), and has been considered as the major bioremediating microorganism in groundwater co-contaminated with these toxic and regulated chemicals (1, 13). In this review, we synthesize the current knowledge regarding *T. lovleyi’s* environmental relevance and bioremediation applications, including its contribution to the nitrogen cycle, reduction of metals and insoluble electron acceptors, and its involvement in the reduction and detoxification of chlorinated organic solvents.

## *T. LOVLEYI* OVERVIEW AND TAXONOMIC CLASSIFICATION

*Trichlorobacter*, alongside *Citrifermentans*, *Geoanaerobacter*, *Geomesophilobacter*, *Geomobilibacter*, *Geomonas*, *Geotalea*, *Oryzomonas*, *Pelotalea*, and *Geobacter*, are genera in *Geobacteraceae* according to the List of Prokaryotes with Standing Nomenclature. *Geobacteraceae* and *Desulfuromonadaceae* were previously families in the order *Geobacterales* according to the National Center for Biotechnology Information (NCBI) (22). However, *Desulfuromonadaceae* has since been reclassified in NCBI as a family in the order *Desulfuromonadales*, while *Geobacteraceae* as a family belonging to *Geobacterales* as of February 2026 (22). The increase in sequencing, genomic research, and taxonomic databases has led to taxonomic reclassification of several members of these families. For example, *Geobacter thiogens* (*G. thiogens*) was initially a species in the genus *Trichlorobacter* (23, 24). *T. lovleyi* and *G. thiogenes* belong to the *Geobacterales* subclade that is capable of respiring organohalides, such as chlorinated solvents (1, 24). *G. thiogenes* can grow on trichloroacetate (24), while *T. lovleyi* respires PCE and trichloroethene (TCE) for its energy metabolism (1).

*T. lovleyi*, originally classified as *Geobacter*, was recently reclassified as *Trichlorobacter*. Its reclassification as *T. lovleyi* was updated in the Genome Taxonomy Database, SILVA SSU rRNA Gene 138 Database, and NCBI but not in the List of Prokaryotic Names with Standing in Nomenclature (25) (Table 1). The established nomenclature by the List of Prokaryotic Names with Standing in Nomenclature was overwritten as *Trichlorobacter lovleyi* (26), contributing to some degree of confusion and lack of cohesion in scientific and academic discourse. For example, a search in Scopus Abstract and Citation Database performed in January 2026 revealed that the nomenclature discrepancy is a relevant issue that needs to be addressed. The search term “*Trichlorobacter lovleyi*” populated 15 results, with the majority being published in 2024 or later, whereas “*Geobacter lovleyi*” populated 260 results published mainly prior to 2022 (27). With more recent literature adopting the nomenclature “*Trichlorobacter lovleyi*” (28–30), it is crucial that information published under the name “*Geobacter lovleyi*” is not lost. At the time of writing this review, the *Trichlorobacter* genus is limited to two species (*lovleyi* and *ammonificans*) according to NCBI. Given the few isolates classified as *Trichlobacter* and the similarity between *T. lovleyi* and *Geobacter* species, this review often compares the physiology and metabolic traits of *T. lovleyi* with *Geobacter*.

**TABLE 1:** Classification of *Trichlorobacter lovleyi* (formerly *Geobacter lovleyi*) strain SZ in different databases^*a*^

	NCBI	SILVA	GTDB	LPSN
Phylum	*Thermodesulfobacteriota*	*Thermodesulfobacteriota*	*Desulfuromonadota*	*Thermodesulfobacteriota*
Class	*Desulfuromonadia*	*Desulfuromonadia*	*Desulfuromonadia*	*Desulfuromonadia*
Order	*Geobacterales*	*Geobacterales*	*Geobacterales*	*Geobacterales*
Family	*Geobacteraceae*	*Geobacteraceae*	*Pseudopelobacteraceae*	*Geobacteraceae*
Genus	*Trichlorobacter*	*Trichlorobacter*	*Trichlorobacter*	*Geobacter*

^*a*^ The database search was performed in February 2026. NCBI, National Center for Biotechnology Information; SILVA, SILVA SSU rRNA Gene 138 Database; GTDB, Genome Taxonomy Database; LPSN, List of Prokaryotic Names with Standing in Nomenclature.

## PERTINENT METABOLIC FEATURES

**Fig 1:**
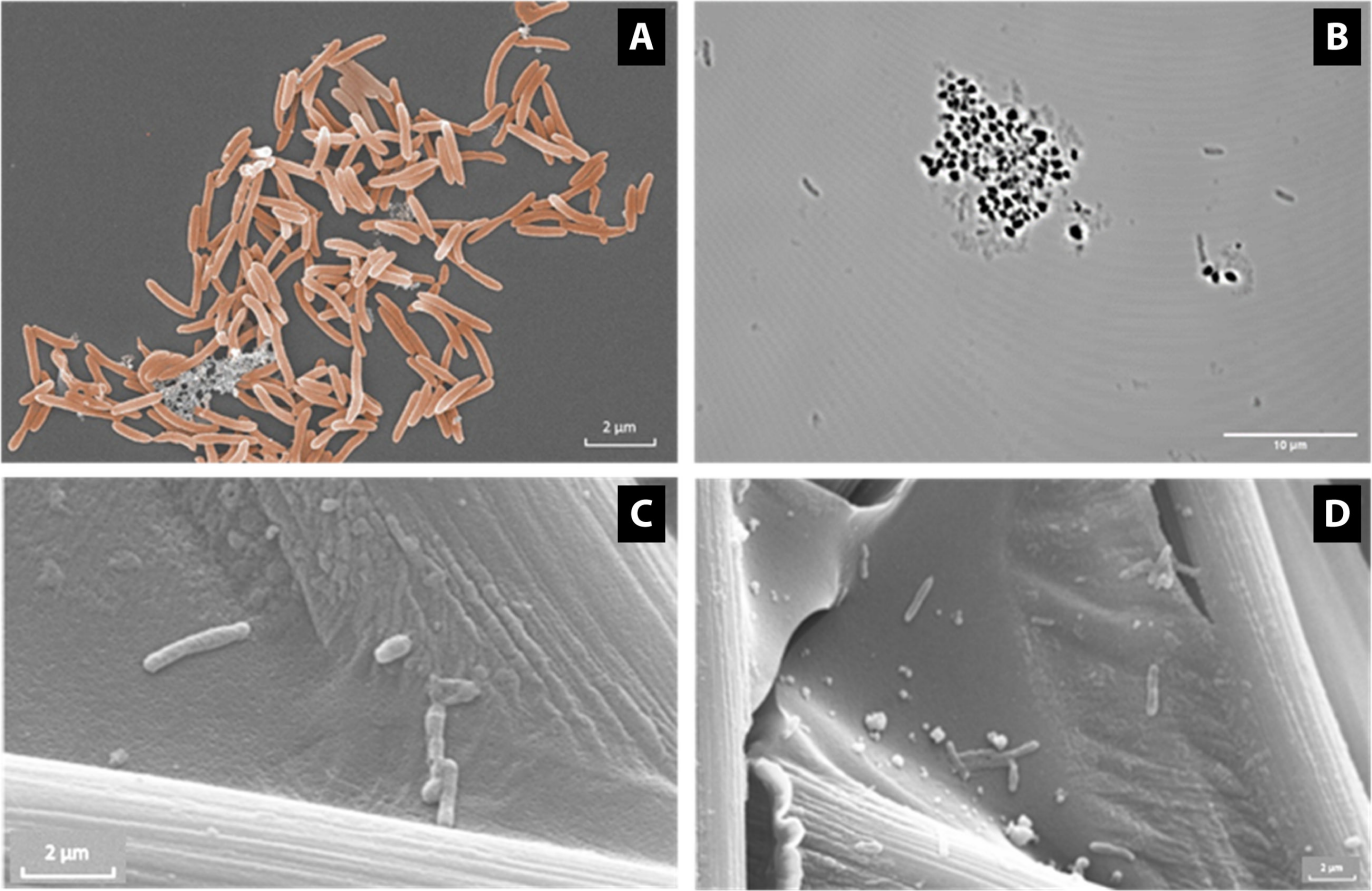
Scanning electron micrographs of *Trichlorobacter lovleyi* (formerly *Geobacter lovleyi*) grown (**A**) planktonically (32) and (**C and D**) attached on carbon paper serving as electrode (33). The image in panel A is courtesy of Shanquan Wang, Provincial Key Laboratory of Environmental Pollution Control and Remediation Technology, Southern Marine Science and Engineering Guangdong Laboratory (Zhuhai), Sun Yat-Sen University, Guangzhou, China. Images in panels C and D are courtesy of Federico Aulenta, Water Research Institute (IRSA), National Research Council (CNR), Rome, Italy, and are republished from reference 33 with permission of the publisher. (**B**) Confocal micrograph of *T. lovleyi* grown planktonically with 0.35 mM U(VI) as uranyl acetate and 10 mM sodium acetate (unpublished data from Delgado lab). Lipid-stained cells (2 µg/mL Nile red) were imaged on Nikon C2+ confocal microscope.

*Geobacter* and *Trichlorobacter* species are well known for their rod-shaped cells with rounded ends (4). The morphology of *T. lovleyi* can be seen in Fig. 1 in a series of micrographs. *Geobacter* and *Trichlorobacter* species are mesophilic anaerobes, mostly chemoorganotrophic, utilizing small organic molecules for energy, though several species use hydrogen as an electron donor if organic carbon sources are also available (1, 31). The sediment from which *T. lovleyi* was first isolated was sampled at Su-Zi Creek near Seoul, South Korea (1). This sediment enrichment was amended with 0.5 mM PCE and 10 mM acetate; PCE was stoichiometrically converted to 1,2-*cis*-dichloroethene (*cis*-DCE) with minimal transient accumulation of TCE (1).

**Fig 2:**
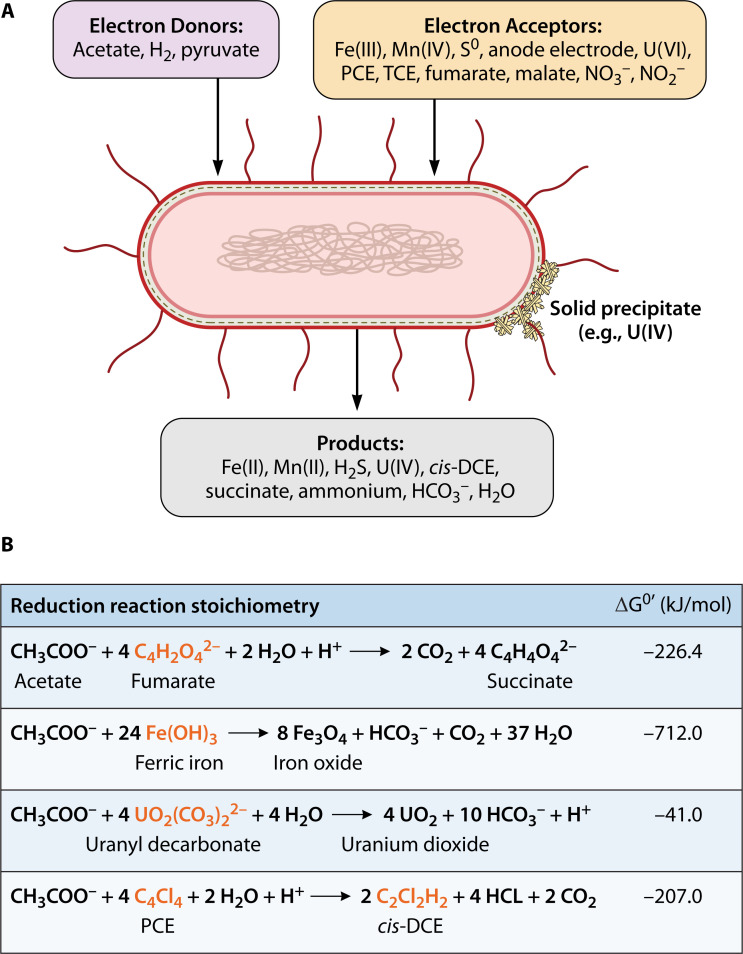
(**A**) Schematic depiction of a *Trichlorobacter lovleyi* (formerly *Geobacter lovleyi*) cell and its metabolic electron donors and acceptors. (**B**) Example stoichiometric reactions and Gibbs-free energy values (*ΔG*^0′^ in kJ/mol acetate) for respiration of fumarate, ferric iron, hexavalent uranium, and PCE. For the stoichiometry of iron reduction, insoluble ferric iron and soluble ferrous iron were assumed. *ΔG*^0′^ values were obtained from these sources (11, 36–42).

Acetate is a common electron donor for *T. lovleyi* (Fig. 2). Acetate oxidation has been extensively studied in *Geobacter/Trichlorobacter* species. Genes encoding for acetate oxidation and assimilation are well conserved in *Geobacter* and *Trichlorobacter* (34). Acetate oxidation and assimilation occur intracellularly, subsequently facilitating the transport of acetate and protons across the inner membrane. Acetate is oxidized to CO_2_ through the TCA cycle. During the TCA cycle, NAD(P)H and reduced ferredoxin are synthesized, which help to provide protons and electrons needed in chemical reactions and biosynthesis. These metabolic products are produced via acetyl-CoA transferase rather than succinyl-CoA synthetase as resolved through metabolic flux and enzymatic assays (35, 36).

**Fig 3:**
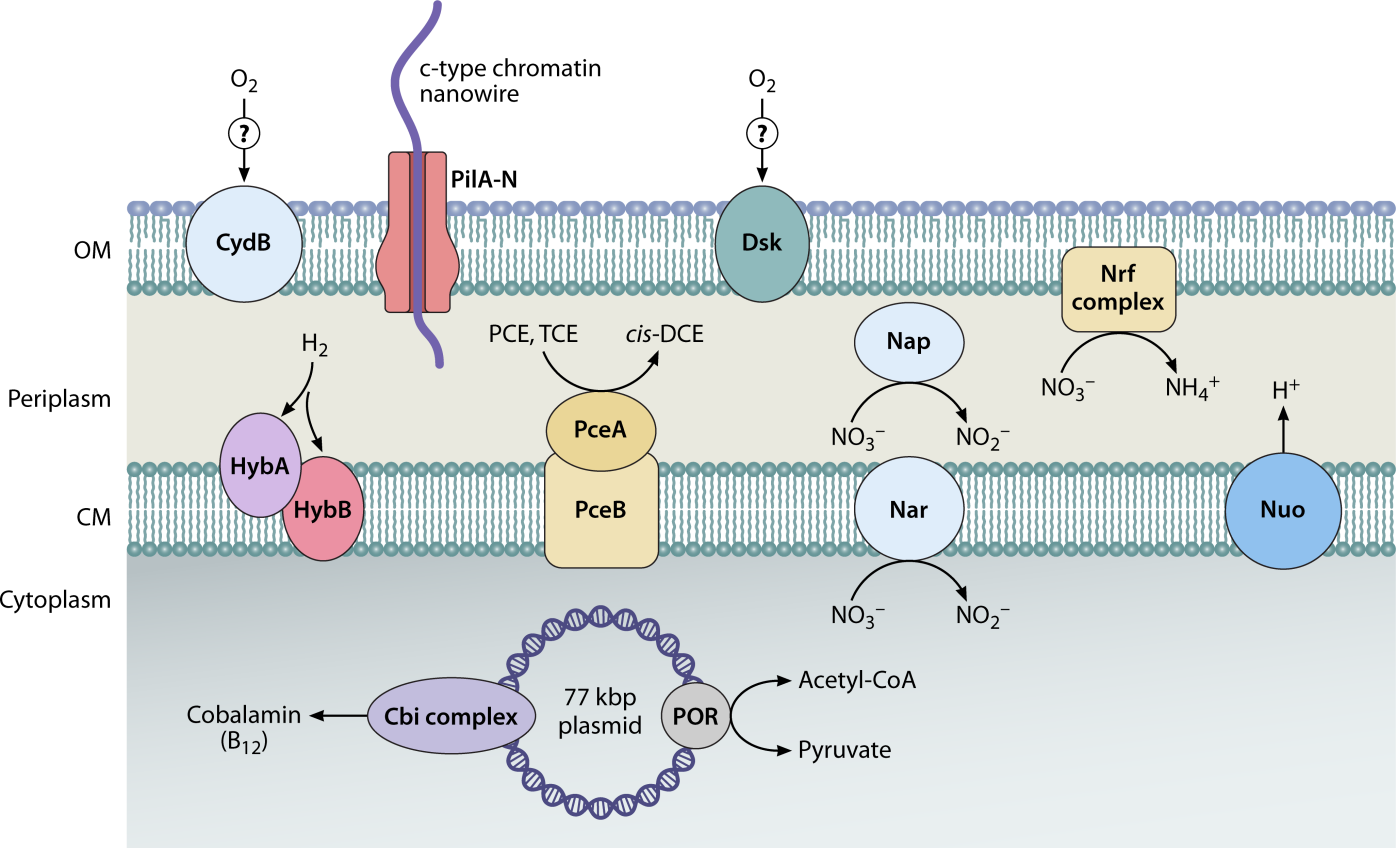
Conceptual model/schematic of the proposed metabolic network in *Trichlorobacter lovleyi* (formerly *Geobacter lovleyi*) involved in key metabolic reactions, including extracellular electron transfer, dissimilatory nitrate reduction to ammonium, pyruvate reduction, hydrogen oxidation, reductive dehalogenation, and cobalamin synthesis. A unique aspect of *T. lovleyi* is the presence of a circular plasmid which encodes for most of the genes involved in cobalamin synthesis. Cbi, cobalamin biosynthesis protein; CydB, cytochrome bd ubiquinol oxidase; Dsk, desulfoferrodoxin; HybA/HybB, hydrogenase complex; Nap/Nar, nitrate reductase; Nrf, nitrite reductase; Nuo, NADH dehydrogenase; PceA/PceB, tetrachloroethene reductive dehalogenase; PilA-N, type IV pilus complex; POR, pyruvate oxidoreductase complex.

Pyruvate (Fig. 2) is a weak electron donor for *Geobacter* and *Trichlorobacter* species, resulting in slower growth rates and cell activity when compared to acetate (43, 44). It has been speculated that pyruvate oxidation is driven by pyruvate oxidoreductase (POR) and a pyruvate dehydrogenase complex (45). The genome of *T. lovleyi* includes five clusters encoding for POR complexes (46). Interestingly, *T. lovleyi* is able to use pyruvate as an electron donor despite missing pyruvate dehydrogenase E1 complex-encoding genes, which can be found in *Geobacter sulfurreducens* (*G. sulfurreducens*), for example (45, 46). It currently remains unclear how *T. lovleyi* oxidizes pyruvate. *T. lovleyi* can use lactate as a carbon source but cannot use it as an electron donor, unlike *G. sulfurreducens*, *Geobacter metallireducens* (*G. metallireducens*), and *Geobacter uraniireducens* (*G. uraniireducens*) (44). In *G. sulfurreducens*, lactate is assimilated *via* lactate oxidase enzyme (44). The genome of *T. lovleyi* encodes for one annotated lactate utilization protein (GLOV_RS14510) (47), which does not facilitate the use of lactate as an electron donor (46, 47). H_2_ oxidation is supported by some members of *Geobacterales* (Fig. 2). In *G. sulfurreducens*, H_2_ is oxidized via an uptake hydrogenase, HybB (6). *T. lovleyi* also encodes for a hybB subunit (GLOV_RS00700) (47) (Fig. 3).

*Geobacter* and *Trichlorobacter* species reduce a number of insoluble electron acceptors (Fig. 2) extracellularly using outer membrane complexes (48). The range of electron acceptors/donors and example stoichiometric reactions relevant to the metabolism of *T. lovleyi* are depicted in Fig. 2. Overall, the metabolic diversity of *T. lovleyi* may provide ecological benefits and explain the occurrence of this microbe in diverse natural and contaminated environments.

The isolate, *T. lovleyi* strain SZ, was obtained through sequential transfers in fresh anaerobic medium and dilution-to-extinction transfers on semisolid medium (1). When grown on fumarate as the sole electron acceptor for multiple successive transfers, *T. lovleyi* can lose its ability to reductively dechlorinate PCE (49). For this reason, *T. lovleyi* cultures require frequent transfers in medium with 0.5–1 mM PCE (50, 51). It is also recommended to minimize air exposure and use reducing agents when cultivating *Geobacter* and *Trichlorobacter* as these microbes are oxygen intolerant (1, 33).

*T. lovleyi* has been characterized as a strict anaerobe, like other *Geobacter* species at their initial discovery. *G. sulfurreducens* and *G. uraniireducens* have mechanisms that allow them to manage oxidative stress (52), and *G. sulfurreducens* can even accept oxygen as a terminal electron acceptor (53). These findings support the common existence of *Geobacter* and *Trichlorobacter* species in subsurface environments where oxygen intrusion is a common occurrence. Cytochrome bd ubiquinol oxidase subunits were the most differentially expressed in *G. uraniireducens* when investigating the stress response to 5% oxygen exposure (52). The cytochrome bd ubiquinol oxidase subunits I and II are involved in the regulation of the RpoS regulon, which is related to growth on oxygen with a high oxygen affinity (54). The genome of *T. lovleyi* includes the cytochrome bd ubiquinol oxidase subunits I (GLOV_RS05940) and II (GLOV_RS05945) (47) (Fig. 3). The presence of these genes hints at the microbe’s ability to handle some level of oxygen, possibly warranting reclassification as an aerotolerant anaerobic bacterium. The genome of *T. lovleyi* also has genes that encode for desulfoferrodoxin (GLOV_RS12820) (47) (shown in Fig. 3). Desulfoferrodoxin is associated with oxidative stress response and was shown to upregulate in *G. sulfurreducens* cultures exposed to 5% oxygen (55).

While *T. lovleyi* lacks many of the oxygen reductases that other *Geobacter* species have (46), there is some evidence to support that this bacterium tolerates small amounts of oxygen (12). The ratio of *T. lovleyi* cells attached to sediment versus unattached was shown to decrease after the introduction of oxygen in a groundwater well at a test site contaminated with U(VI) (12). For microorganisms, attached growth typically aids in mitigating toxicity and better managing environmental perturbations (56, 57). The decrease in the ratio of attached to unattached *T. lovleyi* cells may not necessarily represent a decrease in cell concentrations but rather a shift in the bacterium’s growth preference or adaptive response. Follow-up laboratory experiments with *T. lovleyi* (12) showed no cell growth or acetate consumption in cells exposed to oxygen. It was unclear from the study if fumarate was used to cultivate *T. lovleyi* (12); fumarate is required by other species of *Geobacter* to reduce oxygen (12, 53). The fact that *T. lovleyi* cell concentrations increased in the groundwater *in situ* but not *ex sit*u in laboratory incubations (12) could be indicative of a missing growth requirement or a condition that was not reproduced in the laboratory setup.

Redox potential appeared to be the main factor dictating the metabolic pathways used by *G. sulfurreducens* grown in cell suspensions, outweighing other factors such as electron acceptor type, structure, and solubility (58). When reducing iron citrate, *G. sulfurreducens* uses multiple metabolic pathways to gain a higher energy yield (58). *Geobacter* and *Trichlorobacter* seem to have an adaptive capability to switch their metabolic pathways to fine-tune their metabolism depending on environmental conditions, such as redox potential, to be as efficient as possible (59). Understanding how *T. lovleyi* responds to both the redox potential of electron acceptors/donors and the redox potential gradient in subsurface environments is largely unexplored. Research on this distinctive microbe can enhance our fundamental understanding of how anaerobic microbes respond to changing redox conditions and contribute to biogeochemical cycling in complex environments.

*Geobacter* and *Trichlorobacter* are considered slow-growing microorganisms when compared to some other anaerobes (60) (Table 2). *G. sulfurreducens* grown on acetate and fumarate doubles every 3.7–8.3 h (36, 61, 62). In a recent study, doubling times of 3.9–4.7 h were reported for *T. lovleyi* grown with 20 mM acetate and 20 mM fumarate (28) (Table 2). *T. lovleyi* growth rate depends on the concentration and type of growth-supporting substrate and is affected by product toxicity (e.g., accumulation of *cis*-DCE in the medium when grown on PCE or uraninite in the periplasm when grown on U(VI)). *T. lovleyi* strain SZ is able to reduce 1 mM PCE to an equimolar *cis*-DCE concentration in only 4 days (32). *T. lovleyi* has a reduction rate of approximately 46 nmol L^−1^ min^−1^ for PCE (rate calculated from data in Fig. 2 from reference [1]) and 14 nmol L^−1^ min^−1^ for U(VI) (37). The rates of reduction of PCE and U(VI) were not affected when both electron acceptors were concomitantly utilized by *T. lovleyi* (1); these results indicate that PCE and U(VI) are likely reduced through separate metabolic pathways. Transcriptomic analyses could help elucidate the specific reduction mechanism of PCE and U(VI) by *T. lovleyi*, but such analyses have not been conducted.

**TABLE 2:** Growth rate and doubling time of *Trichlorobacter lovleyi* (formerly *Geobacter lovleyi*) and their comparison to *Geobacter* and other anaerobic species

Species	Substrate(s)	Growth rate (h^−1^)	Doubling time (h)	Reference(s)
*Trichlorobacter lovleyi*	20 mM acetate and 20 mM fumarate	0.16	4.3	(28)
	20 mM acetate and 5 mM nitrate	0.36	1.93	(28)
*Geobacter sulfurreducens*	20 mM acetate and 40 mM fumarate	0.09	7.7	(36)
*Geobacter metallireducens*	15 mM acetate and 20 mM fumarate	0.10	6.6	(34, 63)
*Methanothermobacter marburgensis*	H_2_ and CO_2_ (80%/20%)	0.35	2	(64)
*Bacteriodes fragilis*	45 mM glucose	0.35	2	(65)

## ENVIRONMENTAL RELEVANCE AND BIOREMEDIATION AND BIOENERGY APPLICATIONS OF *T. LOVLEYI*

### Mechanism and implications of dissimilatory nitrate reduction to ammonium

*T. lovleyi* is a contributor to the cycling of nitrogen, especially in terrestrial environments. *T. lovleyi* and *Trichlorobacter ammonificans* perform dissimilatory nitrate reduction to ammonium (DNRA) (3, 7, 8, 66–68). *T. lovleyi* became the dominant microbial species in a chemostat inoculated with activated sludge from a wastewater treatment plant and enriched for DNRA (3). *T. lovleyi* is believed to reduce nitrate via a two-step mechanism where nitrate is first transformed into nitrite, then nitrite is transformed into ammonium in the subsequent step (69). Nitrite is a toxic intermediate that can stall the production of ammonium during DNRA (28). Growth with H_2_ as the electron donor helped to alleviate the toxic buildup of nitrite (28), although carbon sources may need to be supplemented to complete the process of DNRA.

**Fig 4:**
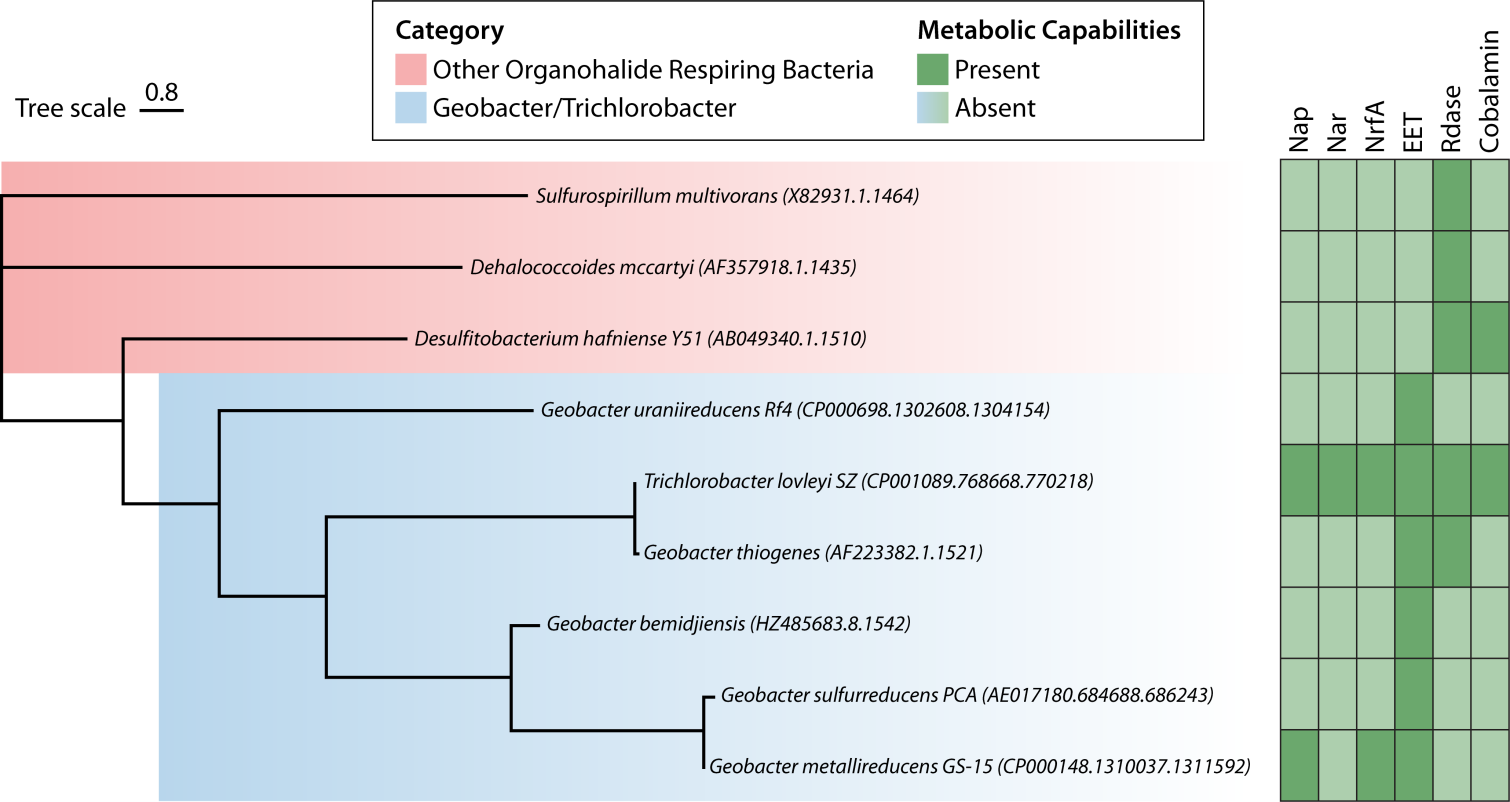
16S ribosomal RNA phylogenetic relationship of *Trichlorobacter lovleyi* (formerly *Geobacter lovleyi*) with other *Geobacter* species and with other organohalide-respiring bacteria co-occurring in enrichment cultures and environmental samples. The 16S rRNA gene sequences were obtained from SILVA database. Phylogenetic relationships were generated with Molecular Evolutionary Genetics Analysis (MEGA) and tvBOT (73).

The first step of DNRA is catalyzed by the nitrate reductase (Nar/Nap), which is found in the membrane and oriented towards the cytoplasm (8). Other DNRA-capable microbes, such as *G. metallireducens*, encode for the Nar complex while *T. lovleyi* encodes for both Nap and Nar, further differentiating *T. lovleyi* (Fig. 3 and 4). This could explain why *T. lovleyi* is more prevalent than other DNRA-capable *Geobacter* species in environments where DNRA is active (28). The second step of DNRA is believed to be catalyzed by the nitrite reductase, NrfA, which is used as a biomarker for DNRA (69, 70). However, some species perform DNRA without the well-known 5-heme NrfA pathway (68). *Trichlorobacter ammonificans* uses a novel Nap-TaNiR complex for DNRA (68). The reduction of nitrate to ammonium in *T. lovleyi* can be differentiated from pathways in other microbes due to its unique cytochrome c nitrite reductase (NrfA) (69). The NrfA enzyme of *T. lovleyi* shares ≤31% similarity to characterized NrfA enzymes (69, 71). This NrfA enzyme has been identified as a part of a specialized DNRA process in *G. metallireducens* and *T. lovleyi*; the enzyme crystallizes as a dimer but is found as a monomer in solution (69, 72). While *T. lovleyi* and *G. metallireducens* both encode for a unique NrfA enzyme, their respective structures can be differentiated by the configuration of the inserted loop region near the active site (72). Furthermore, the NrfA enzyme of *T. lovleyi* does not require Ca^2+^ ions, which are typically part of the active site structure of other characterized NrfA enzymes (69). As bioavailable calcium can be limiting in the environment, Ca-independent NrfA enzymes may provide an advantage to DNRA-performing microorganisms like *T. lovleyi* (69).

While there have been recent clarifications in the mechanisms of DNRA, the major environmental drivers are not well understood. Several studies reported that high C/N ratios select for DNRA, while low C/N ratios select for denitrification (5, 67, 74, 75). Recent research, however, has shown that *T. lovleyi* can perform DNRA at a wide range of C/N ratios given that nitrite concentrations remain below toxic levels (28). Other environmental factors that affect DNRA include environmental redox conditions, with some studies reporting that a low redox potential (e.g., 200–400 Eh) selects for DNRA (74), while others report that a similar potential selects for denitrification (5, 74, 76).

**Fig 5:**
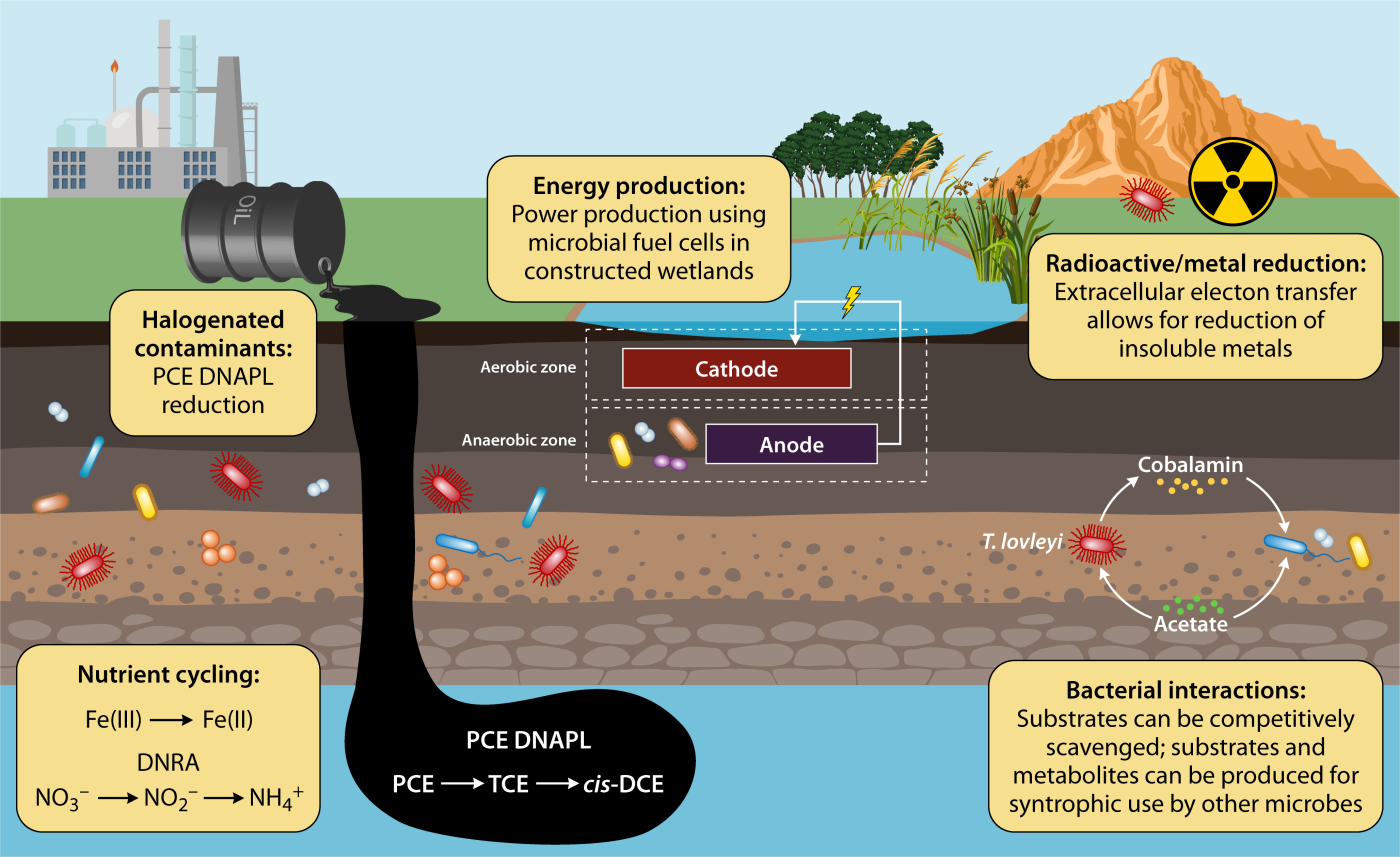
Biogeochemical contributions and engineering applications of *Trichlorobacter lovleyi* (formerly *Geobacter lovleyi*).

Nitrate is a regulated chemical and sometimes a co-contaminant with chlorinated solvents in groundwater and soil (30, 77, 78). The ability to perform DNRA and reductive dechlorination makes *T. lovleyi* a strong candidate for bioremediation applications at impacted sites (Fig. 5). Nitrate can inhibit reductive dechlorination activity and decrease the abundance of some organohalide-respiring bacteria (30, 79). For example, a nitrate concentration of 3 mM led to a decrease in the relative abundance of *Dehalococcoides* (24–19%), while the relative abundance of *Trichlorobacter* (2–11%) increased at this concentration (30). This suggests that *T. lovleyi* is an important functional dechlorinating and nitrate-reducing species with promising potential for bioremediation applications in co-contaminated environments.

### Extracellular electron transfer for bioenergy

The reduction of insoluble metals, such as Fe(III) and Mn(IV), by *Geobacter* and *Trichlorobacter* is enabled by a complex system of conductive protein nanowires *via* direct extracellular electron transfer (EET) (8, 80, 81) (see also Fig. 4). *G. sulfurreducens* is used as a model organism for EET (80–82). EET can be harnessed to produce electricity or generate power with bioelectrochemical and bioenergy technologies. The mechanism of direct EET in *Geobacter* species, a topic of hot scientific debate, is believed to involve electrically conductive nanowires composed of either type IV pilin proteins (81, 83–85) or c-type cytochrome (80, 86–88). On the one hand, it was shown that OmcS facilitated electron transfer from the pili to insoluble compounds (89). However, OmcS was not responsible for the electrical conductivity which was argued to be attributed to pili (89). On the other hand, visualization and characterization studies have identified OmcS and OmcZ cytochromes extruding from *G. sulfurreducens* (82, 90). OmcS was also required for the reduction of insoluble Fe(III) (91). It is likely that *Geobacter* and *Trichlorobacter* can form different types of microbial nanowires based on specific growth conditions (81). Production of multiple types of nanowires is supported by the fact that *G. sulfurreducens* can adapt its EET pathway based on the redox potential of insoluble compounds for its respiratory metabolism (59, 92). The close association between c-type cytochromes and pili is well documented in *G. sulfurreducens*.

C-type cytochromes are not well conserved among EET-performing microbes (34). *T. lovleyi* encodes for 50 cytochromes (46) whereas other *Geobacter* species encode for 70–89 c-type cytochromes (34). Despite having fewer c-type cytochromes and no gene encoding for OmcS or OmcZ, *T. lovleyi* is still capable of many typical functions of *Geobacter*, including Fe(III) reduction. *T. lovleyi* encodes for a putative PilA pilus protein with 81% similarity to the PilA protein of *G. sulfurreducens* (46). While nanowire-like structures extruding from the cell were microscopically observed in cultures grown on Fe(III) (1), the structure and molecular composition of these nanowires have not been investigated.

EET-capable bacteria can be used in microbial fuel cells (MFCs) where they transfer electrons from the oxidation of organic matter generated in the anode to a cathode through an external circuit, generating harvestable energy (16). *Geobacter* and *Trichlorobacter* often dominate in microbial fuel cells used to treat real domestic wastewater (16, 33, 66, 93). MFCs can be combined with constructed wetlands for the treatment of wastewater (MFC-CW). In a study evaluating the energy production of an MFC (average anode potential of −220 mV vs SHE) in a horizontal subsurface constructed wetland treating real domestic wastewater, *Geobacter*/*Trichlorobacter* (97.2% similar to *T. lovleyi*) was highly abundant in the microbial community (93). Different feeds were tested in this study and, interestingly, not all conditions enriched for *Trichlorobacter* (93). The MFCs treating wastewater from the municipal sewer from a settler line had no identifiable *Trichlorobacter* and a lower energy performance than the MFC-CW receiving effluent from a hydrolytic up-flow sludge blanket reactor where *Trichlorobacter* was enriched (93). The MFC-CW system enriched with *Trichlorobacter* species had a maximum current production of 219 mA/m^2^ (93).

The discovery of strains most similar to *T. lovleyi* in an MCF-CW treating real domestic wastewater prompted further investigations in the energy generation and overall performance of *T. lovleyi* in MFCs. *T. lovleyi* appeared to be less efficient at generating energy compared to *G. sulfurreducens* or other *Geobacter* (66). An acetate-fed microbial electrolysis cell (poised at 450 mV vs SHE) colonized by *T. lovleyi* reached a maximum current of 0.09 mA after 10 days, while *G. sulfurreducens* reached a current of 0.64 mA after only 2 days (66). While the potential for *T. lovleyi* to perform EET for bioenergy applications in MFC-CW has been demonstrated (Fig. 5), harvesting *T. lovleyi*’s potential for bioenergy requires more fundamental studies on its EET pathway and dedicated engineering research to optimize EET outputs in bioelectrochemical systems.

### Reductive dechlorination for anaerobic *in situ* bioremediation

*T. lovleyi* is an organohalide-respiring bacterium that can be leveraged for bioremediation of sites contaminated by PCE or TCE (Fig. 5). The chlorinated solvents PCE and TCE are of particular concern due to their negative health impacts, carcinogenicity potential, and disruption to ecological cycles (94). *Geobacter*, most notably strains similar to *T. lovleyi*, are commonly enriched in cultures for the reductive dechlorination of chlorinated solvents (Table 3). *T. lovleyi*’s contribution to the reduction of chlorinated solvents in mixed microbial communities has been documented in laboratory studies (1, 29, 32, 33, 95–97) and field-scale bioremediation applications (19, 20). The relative abundance of *T. lovleyi* (based on DNA amplicon sequencing) is ≤2%, with cell concentrations ranging from 10^6^ to 10^10^ cells/L (Table 3). Note that sources in Table 3 reported *T. lovleyi* as *G. lovleyi*. In some of the tabulated studies, the qPCR biomarkers used were specific to either *Geobacteraceae* or *G. lovleyi* (Table 3), reemphasizing some of the challenges with taxonomic classification and nomenclature of this microbe.

**TABLE 3:** Abundance of *Trichlorobacter* (formerly *Geobacter*) species in selected reductively dechlorinating enrichment cultures used for laboratory studies and bioremediation applications at groundwater contaminated sites*^k^*

Enrichment culture	Culture growth and substrates for reductive dechlorination	*Trichlorobacter^a^* relative abundance (gene copies/L); % ASV or OTU	Source
DehaloR^2	Batch reactor; 1 mM TCE, 5 mM lactate, and 12 mM methanol	2.7 × 10^10*b*,^^*d*^; 0.06^*c*^	(98)
	Semi-batch reactor operated as fill-and draw, 10-day hydraulic retention time; 0.05–0.6 mM TCE, 8 mM lactate, 20 mM methanol	1.1–1.7 × 10^10^^*b*^^,^^*d*^; 1.0-2.0^*c*^	(99)
	Continuous-flow stirred tank reactor, 3-day hydraulic retention time; 2 mM TCE, 10 mM lactate, and 15 mM methanol	3.7 × 10^10^^*a*^^,^^*d*^; n/a	(100)
ZARA-10^*e*^	Batch reactor; 1 mM TCE, 5 mM lactate, and 24 mM methanol	n/a; 0.6^*c*^	(101)
	Flocculated upflow reactor, 3.6 h hydraulic retention time; 2.5 mM TCE, 10 mM lactate, and 15 mM methanol	n/a; 0.8^*c*^	(102)
LINA-09	Batch reactor; 1 mM TCE, 5 mM lactate, and 24 mM methanol	n/a; 0.11^*c*^^,^^*f*^	(101)
MAT-1^*e*^	Batch reactor; 1 mM TCE, 50 mM ethanol, and 50 mM acetate	n/a; 1.0^*c*^	(103)
KB-1^*g*^	Batch reactor; 0.2 mM TCE and 1 mM methanol	30%^*b*^^,^^*h*^; n/a	(104, 105)
Unnamed *Trichlorobacter lovleyi* strain SZ enrichment	Batch reactor containing soil from Ft. Lewis site; TCE, and lactate	4.3 × 10^6^^*b*^^,^^*i*^ ; n/a	(13)
	*In situ* groundwater; TCE [also U(VI)] and ethanol	5.5–6.6 × 10^6^^*b*^^,^^*i*^^,^^*j*^; n/a	(13)

^*a*^ All sources reported the organism’s genus as *Geobacter*.

^*b*^ Relative abundance based on quantitative real-time PCR.

^*c*^ Relative abundance based on DNA amplicon sequencing.

^*d*^ Quantified using primers Geo564F/Geo840R targeting the 16S rRNA gene of *Geobacteraceae* (106).

^*e*^ Developed at Arizona State University and used for field bioremediation applications on a limited scale.

^*f*^ Reported relative abundance of *Geobacteraceae*.

^*g*^ Developed at University of Toronto and commercially produced in SiREM’s facility in Guelph, Ontario. KB-1 is one of the most utilized reductively-dechlorinating cultures for field applications at groundwater contaminated sites.

^*h*^ Quantified using Geo73F/Geo485R primers targeting the 16S rRNA gene of *Geobacter* KB-1 (99% similarity to *T. lovleyi* strain SZ). Amount of gene copies of *Geobacter* KB1 one in culture was reported as a proportion of the total 16S rRNA gene copies of all species. The tabulated value was approximated from Fig. 4 in reference 104.

^*i*^ Quantified using Geo196F/Geo999R primers targeting the 16S rRNA gene of *T. lovleyi* strain SZ.

^*j*^ The tabulated values were estimated from Fig. 2B (impacted) in reference 13.

^*k*^ n/a, not applicable (analysis was not reported or performed).

Historically, organohalide-respiring enrichment cultures have been grown under fermentative conditions using lactate or methanol as major substrates (Table 3). Fermenting bacteria break down lactate or methanol to H_2_ and acetate, fulfilling the electron donor and carbon source requirement for *Dehalococcoides mccartyi* and *Dehalogenimonas,* performing complete reductive dechlorination to ethene, and PCE to *cis*-DCE reductive dechlorinators (e.g., *Trichlorobacter*, *Dehalobacter*, and *Desulfitobacterium*). A recent report demonstrated enrichment of *T. lovleyi* in organohalide-respiring and chain-elongating cultures grown on ethanol and acetate (Table 3). Chain-elongating bacteria convert ethanol and acetate to butyrate, caproate, and caprylate through reverse β-oxidation (107). Chain elongation also produces H_2_. The products of chain elongation serve as secondary sources of H_2_ and carbon for organohalide respiration (103). As dechlorinating cultures similar to those in Table 3 use ferric iron only as a trace nutrient (e.g., at 1.5 mg/L in the growth medium) and ammonium rather than nitrate as a source of nitrogen (108), it is clear that the metabolism of *T. lovleyi* in these communities is organohalide respiration. In KB-1, one of the most utilized reductively dechlorinating cultures for field applications at groundwater contaminated sites, the *Geobacter* species detected (with *Geobacter* biomarker primers Geo73F and Geo485R) had genetic similarity closest to *T. lovleyi* strain SZ (20, 104, 109).

The carbon-chlorine bonds in chlorinated compounds are cleaved by dehalogenase enzymes (110). *T. lovleyi* strain SZ encodes two reductive dehalogenase genes (*rdhA*) (46). The reductive dehalogenase enzyme PceA (Fig. 3) is encoded as a part of a gene cluster of six *pce-*genes located on a chromosomal island in *T. lovleyi* strain SZ (46). The *pce*-gene cluster in *T. lovleyi*, *pceT- pceC-pceA1-pceB1- pceA2-pceB2*, is predicted to be recently acquired through lateral gene transfer based on the lower than average codon usage (46). The two *rdhA* genes encoded by *T. lovleyi* are sufficient in facilitating the reductive dechlorination of PCE to *cis*-DCE. Other *T. lovleyi* strains with 99% similarity to strain SZ have recently been isolated containing only one *rdhA* gene (32). The *rdhA* genes of *T. lovleyi* are also unique in comparison to other organohalide-respiring bacteria, where the typically well-conserved sequential order of these genes differ (111). Despite the *rdhA* genes in *T. lovleyi* having close homology with *Desulfitobacterium* species, its unique *pce*-gene cluster suggests acquisition from an unidentified organohalide-respiring bacterium (111).

The reductive dehalogenase enzymes that catalyze the reductive dehalogenation of chlorinated compounds rely on a cobalamin cofactor (112). Cobamides are a type of corrinoid cofactor that is necessary to catalyze reductive dechlorination reactions by Rdh enzymes (95). Cobamides are composed of an upper and lower ligand connected to a cobalt atom. Cobalamin (vitamin B_12_) is a type of cobamide that is differentiated by its lower ligand, dimethylbenzimidazole (95). While vitamin B_12_ is produced by a variety of microorganisms (113), *T. lovleyi* is one of the few known organohalide-respiring bacterial species that are capable of cobalamin production (46, 114). A unique characteristic of *T. lovleyi* is the occasional presence of a 77 kb plasmid (pSZ77) encoding many of the genes required for cobalamin biosynthesis and membrane transport (46) (Fig. 3). *T. lovleyi* strains that do not possess this plasmid are presumed to carry the genes for cobalamin synthesis in their chromosomal DNA (46). The most similar homologs to the cobalamin genes on pSZ77 are outside *Geobacter/Trichlorobacter*, suggesting that these genes were attained via horizontal gene transfer (46).

During bioremediation efforts, the microbial species, *Dehalococcoides mccartyi* and *Dehalogenimonas* species, are desired because they can completely detoxify the chlorinated solvents, PCE and TCE, to non-chlorinated ethene (115, 116). The obligate organohalide-respiring bacteria *Dehalococcoides mccartyi* and *Dehalogenimonas* rely on external addition or interspecies transfer of a B_12_ cobamide cofactor as they lack genes for production of these corrinoids and rather encode for corrinoid scavenging genes (96) (Fig. 4). In the laboratory, vitamin B_12_ is provided directly to the growth medium in cultures containing *Dehalococcoides mccartyi* and *Dehalogenimonas* (102, 108, 117, 118).

Cocultures of *T. lovleyi* and *Dehalococcoides mccartyi* were able to sustain dechlorination activity without the external addition of cobalamin (vitamin B_12_). On the contrary, cocultures of *G. sulfurreducens* and *Dehalococcoides mccartyi* had negligible dechlorination activity (95). *T. lovleyi* produces a cobamide with dimethylbenzimidazole as the lower ligand which allows *Dehalococcoides mccartyi* to uptake and utilize for reductive dechlorination of TCE to ethene (95). Interestingly, one study reported that dechlorinating enrichment cultures that lacked exogenous vitamin B_12_ yielded a higher abundance of *T. lovleyi* than cultures supplemented with vitamin B_12_ (119). The inability of obligate organohalide-respiring bacteria to synthesize cobalamin makes the syntrophic partnership with cobalamin-producing microbes, such as *T. lovleyi*, a fundamental component in the complete detoxification of chlorinated solvents.

Evidence has shown that a beneficial partnership between *T. lovleyi* and obligate organohalide-respiring bacteria are formed in environments where PCE and TCE are present at inhibitory concentrations. A study investigating the complex cell interactions during dense nonaqueous phase liquid dissolution showed that the highest cell concentration of *T. lovleyi* was measured near the source zone of PCE or TCE, and *T. lovleyi*’s abundance at this location was several magnitudes higher when compared to *Dehalococcoides* (19). The trends in abundance of *T. lovleyi* and *Dehalococcoides* were consistent with observed dechlorination products, where VC was only detected in the plume region downstream of the source zone (19). Investigations into the effect of TCE toxicity in source zones using culture KB-1 revealed that *Dehalococcoides* were mainly responsible for reducing *cis*-DCE under these conditions, while the reduction of TCE to *cis*-DCE was carried out by other microbial species (i.e., *T. lovleyi*) (120). *T. lovleyi* appears to be less impacted by PCE and TCE toxicity in comparison to *Dehalococcoides* species. We therefore argue that, under inhibitory conditions, *T. lovleyi*’s contribution to reductive dechlorination (PCE/TCE to *cis*-DCE) facilitates downstream conversion of *cis*-DCE to ethene by species in *Dehalococcoidia. T. lovleyi* is a meaningful contributor to bioremediation and serves as an ideal syntrophic partner to other organohalide-respiring bacteria through several mechanisms: carries out part of the reductive dechlorination pathway (PCE to *cis*-DCE), reduces the metabolic cost of detoxifying chlorinated solvents, which is pertinent when chlorinated ethenes are at inhibitory concentrations, and provides the necessary cofactor, vitamin B_12_, for the reductive dehalogenase enzymes required for ethene production from chlorinated solvents. *T. lovleyi* also consumes both acetate and H_2_ to low thresholds, promoting fermentation and organic matter oxidation, which replenish H_2_ and organic carbon into the ecosystem.

During *in situ* bioremediation, qPCR biomarkers are commonly employed to track *Dehalococcoides mccartyi* and their reductive dehalogenases. We believe bioremediation efforts at chlorinated ethene-impacted sites could benefit from a similar monitoring for *T. lovleyi* because of their direct participation in contaminant removal and production of vitamin B_12_. We recommend that researchers consider enrichment and culture growth protocols for reductively dechlorinating mixed communities that intentionally increase the abundance of *T. lovleyi*. We conclude that *T. lovleyi* is an important microorganism respiring ferric iron, nitrate, uranium, and chlorinated ethenes with applications for bioremediation and bioenergy. *T. lovleyi* has complex metabolic capabilities and genetic features that have contributed to its convoluted classification. The ubiquitousness of this microbe, its presence and activity in biotechnological systems, and its contributions to biogeochemical cycles and transformation of contaminants warrant further investigation and optimization.
